# The Treatment of Urethral Stricture by Electrolysis

**Published:** 1887-12

**Authors:** 


### *THE TREATMENT OF URETHRAL STRICTURE BY ELECTROLYSIS.*

Robert Newman, M. D., of New York, gives in the *Med. Register* the following *recapitulation* of rules as a safe guide for practitioners who wish to adopt the treatment of electrolysis in strictures of the urethra:

1. Any good galvanic battery will do which has small elements and is steady; the twenty-cell battery, carbon and zinc, is an excellent instrument, sufficient, particularly, for the beginner.

2. The fluid for the battery ought not to be used too strong.

3. Auxiliary instruments, as galvanometer, etc., are important to the expert, but not necessary for the beginner.

4. For the positive pole, a carbon electrode is used, covered with sponge, moistened with hot water, and held firmly against the cutaneous surface of the patient's hand, thigh or abdomen.

5. For the absorption of the stricture, the *negative* pole must be used.

6. Electrode bougies are firm sounds insulated with a hard-baked mass of rubber. The extremity is a mental bulb, egg-shaped, which is the acting part in contact with the stricture.

7. The curve of the bougie is short; large curves are mistakes.

8. The plates must be immersed in the fluid before the electrodes are placed on the patient, and raised again after the electrodes have been removed.

9. All operations must begin and end at zero, increasing and decreasing the current slowly and gradually by one cell at a time, avoiding any shock to the patient.

10. Before operating, the susceptibility of the patient to the electric current should be ascertained.

11. The problem is to absorb the stricture, not to cauterize, burn or destroy tissues.

12. *Weak currents at long* intervals.

13. In most cases, a current of six cells, or from two and a-half to five milliamperes, will do the work; but it must be regulated according to the work to be done.

14. The *seances* should be at intervals, not too frequent in succession.

15. The best position for the patient to assume during the operation is that which is most comfortable for himself and operator. I prefer the erect posture, but the recumbent or others may be used.

16. Anesthetics I like to avoid; I want the patient conscious, so that he can tell how he feels.

17. Force should never be used; the bougie must be guided in the most gentle way; the electricity alone must be allowed to do the work.

18. During one *seance*, two electrodes in succession should never be used.

19. All strictures are amenable to the treatment by electrolysis.

20. Pain should never be inflicted by the use of electrolysis; therefore, it should not be applied when the urethra is in an acute or even sub-acute inflammatory condition.

